# A method for assessing efficiency of bacterial cell disruption and DNA release

**DOI:** 10.1186/s12866-016-0815-3

**Published:** 2016-08-26

**Authors:** Olle M. de Bruin, H. C. Birnboim

**Affiliations:** DNA Genotek, Ottawa, ON Canada

## Abstract

**Background:**

DNA-based testing is becoming the preferred method both for identifying microorganisms and for characterizing microbial communities. However, no single DNA extraction method exists that is suitable for all types of microorganisms because bacteria are variable in their susceptibility to lysis by available extraction procedures. To develop and test new DNA extraction procedures, it would be helpful to determine their efficiencies. While the amount of extracted DNA can readily be measured by different methods, calculation of true efficiency requires knowledge of the initial amount of DNA in the starting bacterial sample, which cannot be done with precision by any existing method. In the process of developing a new extraction procedure, we developed a method that can be used to determine the total amount of both DNA and RNA in bacteria. The amount of DNA can be calculated from the amount of purines released after mild acid and alkali treatment. The amount of RNA in the same extract can also be calculated from the amount of ribonucleoside monophosphates. The released purines and ribonucleoside monophosphates can be quantified by absorbance using HPLC, with reference to appropriate standards.

**Results:**

The acid/HPLC method was used to measure the efficiency of commonly used bead-beating and chemical protocols for releasing DNA from a particularly hardy organism, *Mycobacterium smegmatis* as well as several other species (*Bacillus subtilis* vegetative cells and spores; *Francisella philomiragia*; *Pseudomonas aeruginosa; Moraxella catarrhalis; Bacillus thuringiensis*; *Staphylococcus aureus*). Surprisingly large differences in efficiency between methods were found.

**Conclusions:**

The acid/HPLC method is a new tool to determine DNA extraction efficiencies and should aid in the development of improved protocols for releasing DNA from a broad range of microorganisms.

**Electronic supplementary material:**

The online version of this article (doi:10.1186/s12866-016-0815-3) contains supplementary material, which is available to authorized users.

## Background

Nucleic acid testing (NAT) is an important alternative to traditional culture methods for identifying microorganisms of clinical relevance. Culture methods take days to weeks to provide clinically useful information whereas nucleic acid testing can provide information, such as strain and drug resistance, in few hours [1]. Sensitivity of NAT can potentially match culture methods if DNA of sufficient quantity (and purity) can be recovered from the biospecimen. The quantity of DNA may be limiting in cases where the number of microorganisms in the original biospecimen is very low or where the volume of the specimen is small.

An additional factor that affects the quantity of DNA available for analysis is the efficiency of DNA release by the extraction procedure from bacteria or other microorganisms. For example, it is especially difficult to release DNA from bacteria such as *Mycobacterium* spp. To extract DNA from such microorganisms, a combination of physical (heat), mechanical (sonication, bead-beating) and/or chemical (pH, detergents) methods have been used [2–8]. However, studies of this type have all lacked a precise way to determine the efficiency of DNA release. High efficiency of DNA release may be expected to be particularly important in microbiome research, since profiling would benefit from obtaining DNA from all microorganisms equally in a biospecimen. Without equal efficiency of extraction, microbes that are hard to lyse may be underrepresented [2, 9]. High efficiency of DNA extraction is also desirable for NAT-based diagnosis of infectious diseases caused by difficult-to-lyse bacteria such as *Mycobacterium* spp.*,* and for identification of bio-threat agents, including bacterial spores [10, 11].

To calculate the efficiency of extraction, the amount of DNA recovered and the total amount of DNA in the original sample must both be known. There is currently no generally accepted method for accurately quantifying the amount of DNA in a suspension or pellet of intact bacteria. Indirect methods for estimating cellular DNA content are based on microscopic cell counts or colony counts. However, these require accurate knowledge of the amount of DNA per cell, which is difficult to assess because the amount of DNA varies with the number of genome copies, which in turn is a function of growth rate. Counting of cells is non-trivial since many bacteria have a tendency to form clumps or chains. For these reasons, it has been very difficult to compare the efficiency of DNA released by different published extraction procedures.

In this report, we describe an acid/HPLC method that uses simple chemical principles to estimate the amount of DNA and RNA in a suspension of microorganisms (see diagram below) and compare it to existing methods. Our method is based upon selective acid-catalysed depurination of DNA [12, 13]. The quantity of DNA can be calculated from the quantity of purines released, provided depurination of DNA is complete and depurination of RNA is very low. Estimation of RNA in the same sample is also possible. Treatment of RNA under mild alkaline conditions causes its degradation to ribonucleoside monophosphates, with no degradation of DNA. Purines released from DNA and ribonucleoside monophosphates from RNA can be quantified by absorbance using HPLC, using appropriate reference standards.
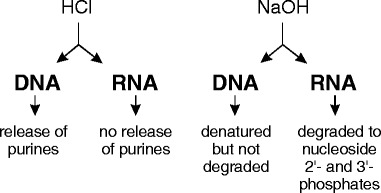


We present evidence that the acid/HPLC technique can provide an accurate measure of the total amount of DNA in a bacterial sample, allowing a comparison of DNA release efficiency by any of the DNA extraction protocols in current use. We report surprisingly large differences between different cell disruption techniques. Although our method was developed primarily to quantify DNA in bacteria, the technique should be also applicable to other types of biological samples.

## Methods

### Reagents

Materials were purchased from the indicated suppliers as follows: Canine DNA (EMD Millipore, Billerica, MA, USA); adenosine 2’(3’)-monophosphate mixed isomers, adenosine 3’-monophosphate, lysozyme, pluronic F-68, reduced Triton X-100, poly(A) and yeast RNA (ribonucleic acid type VI) (Sigma); ADA buffer, guanine and adenine (Alfa Aesar); guanidine hydrochloride (Amresco, Solon, OH, USA). Yeast RNA was treated with deoxyribonuclease to remove traces of DNA. 1.0 N HCl and 1.0 N NaOH were from Ricca Chemical. CDTA (cyclohexanediamine tetraacetate) was from GFS Chemicals.

### Bacteria and growth conditions

*Mycobacterium smegmatis* (Trevisan) Lehmann and Neumann (ATCC 700044), *Bacillus subtilis, Bacillus thuringiensis* (ATCC 10792), *Pseudomonas aeruginosa* (ATCC 10145) and *Staphylococcus aureus* were grown on tryptic soy agar (TSA) plates at 37 °C. Liquid cultures of bacteria were grown in tryptic soy broth (TSB) at 37 °C on a shaking incubator. *Moraxella catarrhalis* (ATCC 25238) was grown on brain heart infusion agar and broth at 37 °C. *Francisella philomiragia* (ATCC 25017) was grown on TSA plates supplemented with 0.1 % cysteine and in TSB supplemented with 0.1 % cysteine at 37 °C.

### Spore preparation

*B. subtilis* spores were grown in 1/10th strength Columbia broth (Difco, Sparks, MD, USA), supplemented with 100 μM manganese sulfate, for 3 days at 37 °C, as described by others [14]. Spores were harvested, washed and incubated with lysozyme at 37 °C for 1 h to remove residual vegetative cells. Weakened cells were lysed in 0.1 % sodium dodecyl sulfate (SDS). Following another wash step, DNA attached to spore cell walls was removed by treatment with DNase (10 μg/ml in 4 mM MgCl_2_, 1 mM CaCl_2_, 10 mM Tris–HCl, pH 7.5). DNase was inactivated at 75 °C for 10 min, and purity of the spore preparation was verified by microscopic examination following Schaeffer-Fulton staining [15].

### Standard Acid/Alkali-treatment of bacterial cells and extracts

Bacteria from liquid or agarose plate cultures were washed by centrifugation in cold phosphate-buffered saline (PBS) (137 mM NaCl, 2.7 mM KCl, 10 mM sodium phosphate, 1.8 mM potassium phosphate, pH 7.4). Washed pellets were well-suspended in 320 μl of water; 80 μl of 1.0 N HCl was then added and the suspension mixed. Samples were incubated for 60 min at 60 °C in a water bath, vortexing at 0, 30 and 60 min. To each sample, 133.3 μl of 1.0 N NaOH was added (80 μl to neutralize the HCl and 53.3 μl to bring the final concentration to 0.1 N). Samples were heated at 100 °C for 10 min to hydrolyze RNA to ribonucleoside 2’- and 3’-monophosphates. Samples were centrifuged at 20,817 *g* for 5 min to remove insoluble material. A portion (400 μl) of each supernatant was removed. Samples were brought to neutral pH by addition of 40 μl of 1.0 N HCl and 160 μl of 0.4 M ADA buffer, pH 6.6, following which they were loaded onto a HPLC column.

### HPLC

Samples were analyzed using a Perkin Elmer HPLC system comprising a series 200 UV/VIS detector, a series 200 pump and a series 225 autosampler. The column was a reverse phase Gemini-NX-C18 with 3 μm particles (Phenomenex, Inc.). The isocratic solvent contained 2.0 % (w/v) methanol in 30 mM ammonium acetate, 1 mM CDTA, 10 mM NaH_2_PO_4_, pH 6.3. Pump speed was 0.5 ml/min, and detector wavelength was 260 nm. Up to 40 μl of sample could be injected, depending upon the expected concentration of the analyte of interest. Area under the curve (AUC) was calculated using TotalChrom Navigator version 6.3.2 software (Perkin Elmer). AUC was converted to amount of analyte from a standard curve of the analyte of interest.

### Preparation of adenine HPLC standards

A solution of adenine used as a standard for HPLC was prepared as follows. Approximately 2.5 mg of adenine (Alfa Aesar) was mixed with 100 mL of 10 mM HCl. The mixture was heated at 50 °C for 60 min, then filtered through a 0.22 μM membrane. The concentration of adenine was calculated using 13,200 as the molar extinction coefficient at 262.5 nm under acidic conditions [16]. Serial dilutions in 10 mM HCl were prepared as required.

### Calculation of DNA and extraction efficiency from the amount of adenine or adenine + guanine

In this report, we focus on pure cultures of microorganisms whose DNA base composition is known. The quantity of adenine released from the cells by treatment with acid and alkali, as described above, can be used to calculate the quantity of DNA present in cells. However, if the base composition is not known or if a mixed population of cells of different base compositions is analyzed, the quantity of *both adenine and guanine* must be determined to calculate the amount of DNA. A spreadsheet for converting nanomoles or nanograms of adenine or adenine + guanine to nanograms of DNA is provided as Additional file 1. Under our defined conditions (see Methods), guanine peaks appear at the 6 min mark of HPLC profiles of acid/alkali treated samples (see Fig. 3), and the amount of guanine can be determined using guanine standards of known concentration. Once the amount of DNA in samples prior to and after treatment with a lysis method has been calculated (either using adenine or adenine + guanine), the extraction efficiency of a lysis method then equals the amount of DNA released from the mixed population by the lysis method divided by the total amount of DNA in the mixed population.

### Bead-beating

Two ml of a stationary phase culture of bacteria, approximately 2 × 10^9^ cells/ml, was harvested by centrifugation, washed twice and resuspended in 1.5 ml of cold PBS. The suspension was distributed equally into three screw-cap polypropylene 1.5 ml vials (Simport, Beloeil, Quebec, Canada) containing approximately 100 μl of 100 μm glass beads (Polyscience, Inc., Warrington, PA, USA). Bead-beating was performed in a Mini-Beadbeater-16 (Biospec) in two cycles of 1 min at 3,450 oscillations/min, with a 1 min period of cooling on ice between cycles. Each sample was transferred to a fresh 1.5 ml microcentrifuge tube and centrifuged at 20,817 *g* for 5 min to remove unbroken cells and debris. 320 μl of the supernatant was removed and treated with standard acid/alkali, as described above.

### Enumeration of bacteria by plate counting

*Bacillus subtilis* were grown in tryptic soy broth to mid-logarithmic phase and harvested by centrifugation. Cells were washed twice in cold tris-buffered saline and divided equally into six tubes. Three aliquots were serially diluted into TSB and plated on TSA. After incubation for 18 h at 37 °C, colony-forming units (CFUs) were enumerated. Bacterial pellets in the remaining three tubes were treated with standard acid/alkali as described above. Plate counting to enumerate *M. smegmatis* followed a similar procedure. CFU counts were converted to DNA content for comparison to acid/HPLC results based upon reported genome sequences (http://www.ncbi.nlm.nih.gov/genome/). For *B. subtilis*, its genome size is 4.2 Mb, which corresponds to 4.9 μg/10^9^ cells; for *M. smegmatis*, its genome size is 7.1 Mb, which correspond to 8.2 μg/10^9^ cells.

### Lysis of vegetative *Bacillus subtilis* cells with lysozyme and SDS

*B. subtilis* was grown to exponential phase in LB broth and harvested by centrifugation. Cells were washed and resuspended in ice-cold TE. The suspension was divided equally into tubes and again centrifuged. Two of the pellets were suspended in 950 μl TE containing lysozyme (1 mg/ml), followed by incubation for 45 min at 37 °C. SDS (final concentration of 0.5 %) was added to ensure complete lysis of the cells. KCl (0.1 N) was added; after incubation on ice for 5 min, the precipitate was removed by centrifugation at 20,817 *g* for 10 min. An aliquot of the supernatant was transferred to a fresh tube and subjected to standard acid/alkali treatment. The remaining two pellets were suspended directly in 500 μl water and subjected to standard acid/alkali treatment.

### DNA extraction from *M. smegmatis*

*M. smegmatis* were scraped from four TSA plates, washed twice in ice-cold water and suspended in 50 mM tris pH 8, 1 mM CDTA. For bead-beating in the presence of detergent, SDS or pluronic F-68 was added to a final concentration of 1.0 %. Bead-beating was performed as described above. Boiling was performed in a water bath for 10, 20 or 30 min. Freeze-boil was performed by placing tubes containing bacterial suspensions at −20 °C for 10 min, followed by heating in a boiling water bath for 10 min. Following each treatment, all samples were cleared by centrifugation for 5 min at 20,817 *g*, and then subjected to standard acid/alkali treatment.

To test the effectiveness of guanidine hydrochloride as a DNA extraction reagent, a previously described procedure was followed [17]. *M. smegmatis*, scraped from TSA plates, was washed three times with cold PBS and distributed equally into 12 tubes. Pellets were suspended in triplicate in 200 μl of either (i) 8 M guanidine hydrochloride, 2 % reduced triton X-100, 80 mM Tris–HCl, pH 8.0, 40 mM CDTA, (ii) 2 % reduced triton X-100, 80 mM Tris–HCl, pH 8.0, 40 mM CDTA or (iii) water, then heated at 100 °C for 10 min. After heating, samples were centrifuged at 20,817 *g* for 5 min to remove unbroken cells and debris. Following centrifugation, the supernatant was removed and subjected to standard acid/alkali treatment as described above. Standard acid/alkali treatment of the untreated cell pellets was also carried out. The remaining three pellets were subjected directly to standard acid/alkali treatment.

## Results

### Selective release of adenine from DNA as compared to RNA

**Fig. 1:**
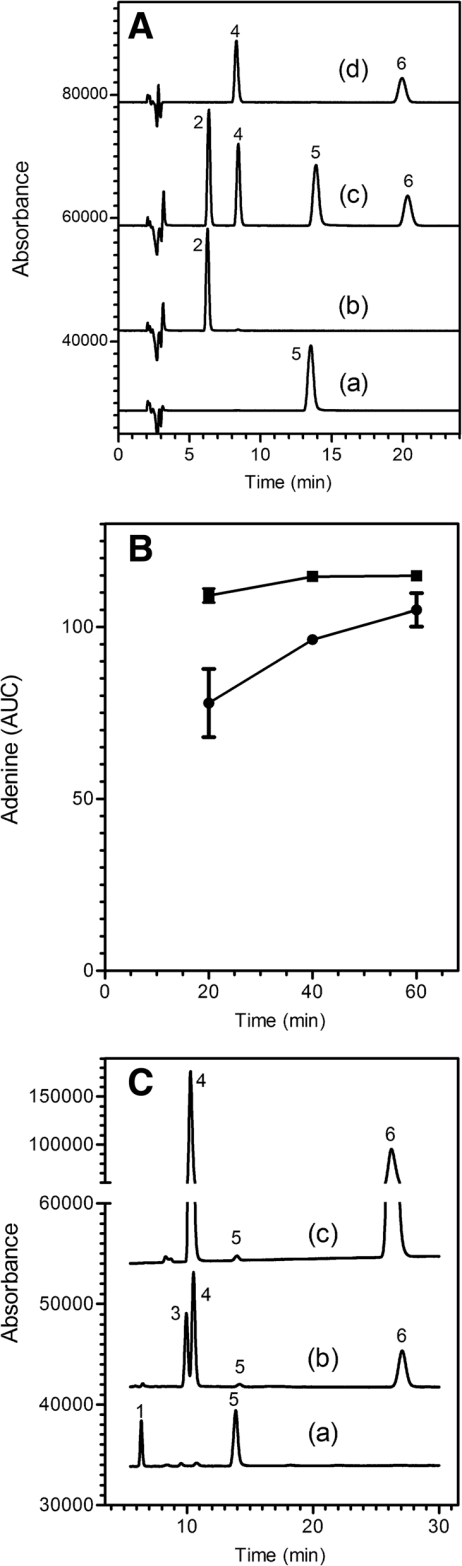
(**a**). Separation of purines and ribonucleoside monophosphates by reverse phase HPLC. Curve (*a*), adenine; curve (*b*), guanine; curve (*c*), mixture of adenine, guanine, adenosine 2’-phosphate and adenosine 3’-phosphate; curve (*d*), mixture of adenosine 2’-phosphate and adenosine 3’-phosphate. (**b**). Release of adenine from pure DNA by acid hydrolysis. The DNA was incubated in 0.15 N or 0.20 N HCl at 60 °C for 20, 40 or 60 min, then the amount of released adenine was quantified by HPLC. The quantity of DNA (calculated from the amount of adenine) was compared to the amount specified by the manufacturer (Novagen). For the 0.15 N treatment (solid circle), the amounts were 34.3, 86.7 and 94.5 %, respectively. For the 0.20 N treatment (solid square), the amounts were 99.3, 103.3 and 103.3 %, respectively. Error bars represent range of duplicate samples; where not shown, the values are within the symbol. (**c**). Selective release of adenine from DNA compared to RNA. DNA (curve a), RNA (curve b) and poly(rA) (curve c), were subjected to standard acid/alkali treatment, then analysed by HPLC. The expected peaks of guanine and adenine from DNA, adenosine 3’-phosphate and adenosine 2’-phosphate from poly(rA) and adenosine 3’-phosphate, adenosine 2’-phosphate and guanosine 2’-phosphate from RNA can be seen. In addition, tiny peaks of adenine can be seen in the poly(rA) and RNA samples, representing 0.25 and 1.63 % of the total adenosine phosphate from poly(A) and yeast RNA, respectively. Peaks in this and subsequent figures are identified by the following numbers: *1*, adenosine 5’-phosphate; *2*, guanine; *3*, guanosine phosphate; *4*, adenosine 3’-phosphate; *5*, adenine; *6*, adenosine 2’-phosphate. The guanosine phosphate isomer (Peak *3*) is likely to be the 2’-phosphate, based on the order of elution of the adenosine monophosphates in our isocratic HPLC system and of the guanosine and adenosine phosphates on an ion-exchange system [22]. Absorbance at 260 nm in milliabsorbance units; adenine (AUC), Area Under the Curve for adenine in milliabsorbance units

DNA is degraded on exposure to relatively mild acidic conditions in two separate steps. First, purines are readily released from deoxyribose while pyrimidine-deoxyribose linkages are highly resistant. Subsequently, the resultant apurinic acid is cleaved by β-elimination to yield a series of pyrimidine isostichs [12, 13, 18]. Alkali treatment of intact, double-stranded DNA causes denaturation without degradation, but generates ribonucleoside monophosphates from RNA. Purines (adenine and guanine) and adenine ribonucleoside monophosphates (2’-AMP and 3’-AMP) can be separated and quantified by isocratic HPLC (Fig. 1a).

To determine conditions of acid treatment that produce complete depurination of DNA without depurination of RNA, we subjected DNA to treatment with either 0.15 N or 0.20 N HCl at 60 °C for periods up to 60 min. Release of adenine was quantified by HPLC. The data presented in Fig. 1b shows that near-maximum release of adenine occurred at 60 min in 0.15 N HCl and that maximum release of adenine occurred at 40 min and 60 min in 0.20 N HCl. We chose 0.2 N HCl for 60 min at 60 °C as standard acid treatment for all subsequent experiments.

We next determined the effect of the same acid conditions on the release of purines from RNA. Yeast RNA and poly(rA) were treated with standard acid and alkali conditions and analyzed by HPLC (Fig. 1c). A very small peak of free adenine can be detected, which represents 0.25 % of the total adenine nucleotide from poly(rA) and 1.63 % from yeast RNA. These experiments demonstrate that, under the conditions described, release of purines from DNA is virtually complete while <2 % of purines are released from RNA. This difference is the basis for our acid/HPLC method to quantify DNA. Knowing the quantity of adenine (or adenine + guanine) allows calculation of the amount of DNA in a sample (see Methods and Additional file 1.

To quantify RNA as well as DNA, we introduced an alkali treatment step after the acid treatment step to complete the partial hydrolysis of RNA (e.g., oligoribonucleotides, cyclic 2′,3’-nucleoside monophosphates) to ribonucleoside 2’-monophosphates and ribonucleoside 3’-monophosphates. The standard acid/HPLC protocol, as described in the Methods, includes both an acid step and an alkali step.

### Comparison of acid/HPLC with other methods used to quantifying the amount of DNA in a sample of bacteria

As indicated above, there is no standard method for estimating the total amount of DNA in intact bacteria. We therefore compared our acid/HPLC method with three other methods that might be used for estimating the total amount of DNA in a sample of intact bacteria. The first two methods involve disrupting cells with either lysozyme (an enzyme suitable for gram-positive cells) or bead-beating (mechanical disruption by vigorous shaking with glass beads). Assuming complete disruption of the cells, released DNA can then be measured and the total amount of DNA estimated. For example, lysozyme treatment followed by SDS is a very effective disruption method of vegetative *B. subtilis* cells that should lead to release of all, or nearly all, DNA in the bacteria. A third way of estimating the amount of DNA in a sample of bacteria is enumeration of cell number using viable colony counts.

**Fig. 2:**
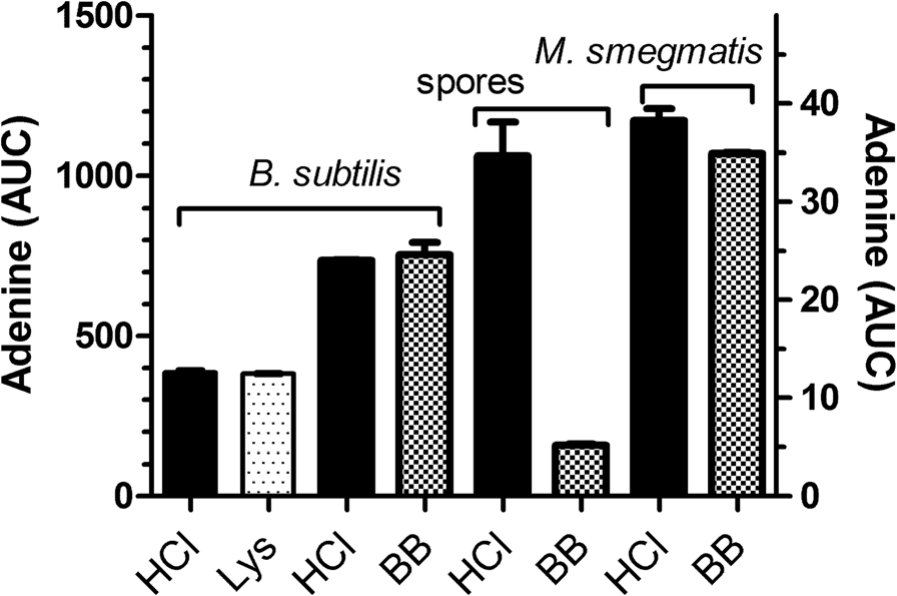
Comparison of the amount of DNA in intact cells, as detected by acid/HPLC, with the amount of DNA released after different disruption methods. Disruption methods used were lysozyme (Lys) for *B.*vegetative cells and bead-beating (BB) for *B. subtilis* spores and *M. smegmatis*. In each experiment, an aliquot of a cell suspension(6x10^8^ *B. subtilis* vegetative or 8x10^8^ *B. subtilis* spores, or 2x10^9^ cells of *M. smegmatis*) was treated either by (i) the standard acid/alkali method (HCl) or (ii) disrupted as indicated. After disruption, lysates were centrifuged to remove debris, then treated by the standard acid/alkali method to release adenine from the released DNA. The amount of adenine was determined by HPLC. The mean and range of duplicate samples are shown. The right-hand axis refers to the spores samples. Adenine (AUC), Area Under the Curve for adenine in milliabsorbance units

The amount of DNA present in bacterial suspensions of *B. subtilis* and *Mycobacterium smegmatis* as determined directly by the acid/HPLC method is shown in Fig. 2. These values were compared to the amount of DNA released from disrupted cells. Disruption by lysozyme treatment was used for vegetative *B. subtilis* cells and bead-beating was used for *M. smegmatis* and *B. subtilis* (vegetative cells and spores). After centrifugation to remove debris and any unbroken cells, the amount of DNA in the supernatant of the disrupted cells was measured using acid/HPLC. In Fig. 2, ‘HCl’ represents the amount of DNA in ‘intact’ bacterial cells, measured directly using acid/HPLC. The results show that, in all cases except spores, the amount of DNA detected in intact cells by acid/HPLC was (within experimental error) the same as the amount of DNA released from cells first completely disrupted by lysozyme or bead-beating. For spores, acid/HPLC detected about seven times more DNA than detected in the cell supernatant after bead-beating (Fig. 2). Bacterial spores are known to represent a particularly difficult challenge for release of DNA [19, 20]. These results support the notion that the direct acid/HPLC method is detecting essentially all DNA in these microorganisms.

Plate counting was the third method tested as a comparison with acid/HPLC. To convert colony counts (CFUs) to total DNA requires knowledge of the amount of DNA per cell, as calculated from the genome sequence of the target organism. For *B. subtilis*, its genome size is 4.2 Mb, which corresponds to 4.9 μg/10^9^ cells; for *M. smegmatis*, its genome size is 7.1 Mb, which correspond to 8.2 μg/10^9^ cells (http://www.ncbi.nlm.nih.gov/genome/). Table 1 shows DNA content estimated from colony counts compared to DNA measured by acid/HPLC. In both cases, the estimated amount by CFU is less than the measured amount (81 % for *B. subtilis* and 38 % for *M. smegmatis*). A spread sheet for converting nanomoles or nanograms of adenine to nanograms of DNA is provided as Additional file 1 (see also Methods section).

**Table 1:** Comparison of CFU and acid/HPLC to quantify the amount of DNA in bacteria

Microorganism	DNA content (μg) based on CFU	DNA content (μg) based on acid/HPLC
*B. subtilis*	1.32 ± 0.034 (*n* = 3)^a^	1.62 ± 0.029 (*n* = 3)
*M. smegmatis*	6.10 ± 0.906 (*n* = 5)^b^	15.91 ± 0.338 (*n* = 5)

^a^Assumes that *Bacillus subtilis* contains 4.9 μg DNA/10^9^ cells

^b^Assumes that *Mycobacterium smegmatis* contains 8.2 μg DNA/10^9^ cells

### Examples of acid/alkali-treated extracts of *B. subtilis*, *E. coli*, *M. smegmatis* and baker’s yeast

**Fig. 3:**
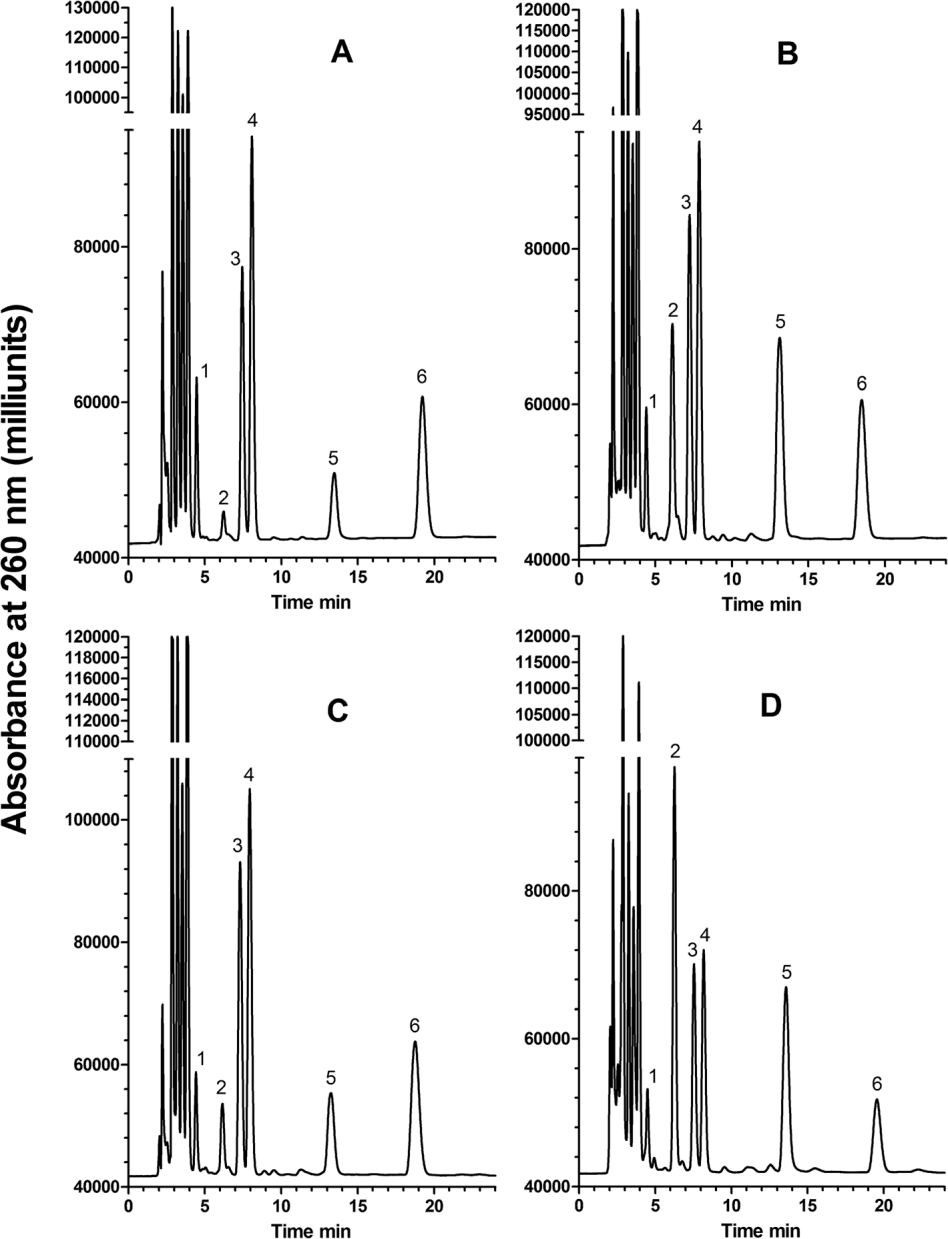
Examples of HPLC profiles of acid/alkali extracts of four different types of microorganisms. Washed cell pellets, prepared as described in the Methods, were subjected to the standard acid/HPLC protocol. **a** *S. cerevisiae* (baker’s yeast); **b** *E. coli* (gram-negative bacterium); **c** *B. subtilis* (gram-positive bacterium); **d** *M. smegmatis,* (bacterium with a waxy cell wall)*.* Numbers near the peaks refer to the identity of known components, listed in the legend to Fig. 1

Figure 3 shows HPLC profiles obtained after acid/alkali treatment of pure cultures of microorganisms. Qualitative information about the nucleic acids in each organism can be obtained from inspection of the profiles. For example, the ratio of peak 5 (adenine) and peak 6 (ribonucleoside 2’-monophosphate) reflects the approximate relative amount of DNA and RNA, respectively, in the four different microbial species. Of the organisms tested, *M. smegmatis* had the lowest RNA/DNA ratio, *E. coli* had a slightly higher ratio and *B. subtilis* and *S. cerevisiae* had the highest ratios. The GC content of the different microorganisms can also be estimated from the relative guanine (peak 2) to adenine (peak 5) ratio. Reported GC contents for these organisms are 43.5 % for *B. subtilis*, 38.2 % for *S. cerevisiae*, 50.8 % for *E. coli* and 66.5 % for *M. smegmatis* (www.ncbi.nlm.nih.gov/genome/). Although a precise GC content was not determined in this experiment, the high gua/ade ratio of *M. smegmatis* can be seen in panel D.

### Differences in the amount of DNA released from *Mycobacteria smegmatis* by different extraction methods

**Fig. 4:**
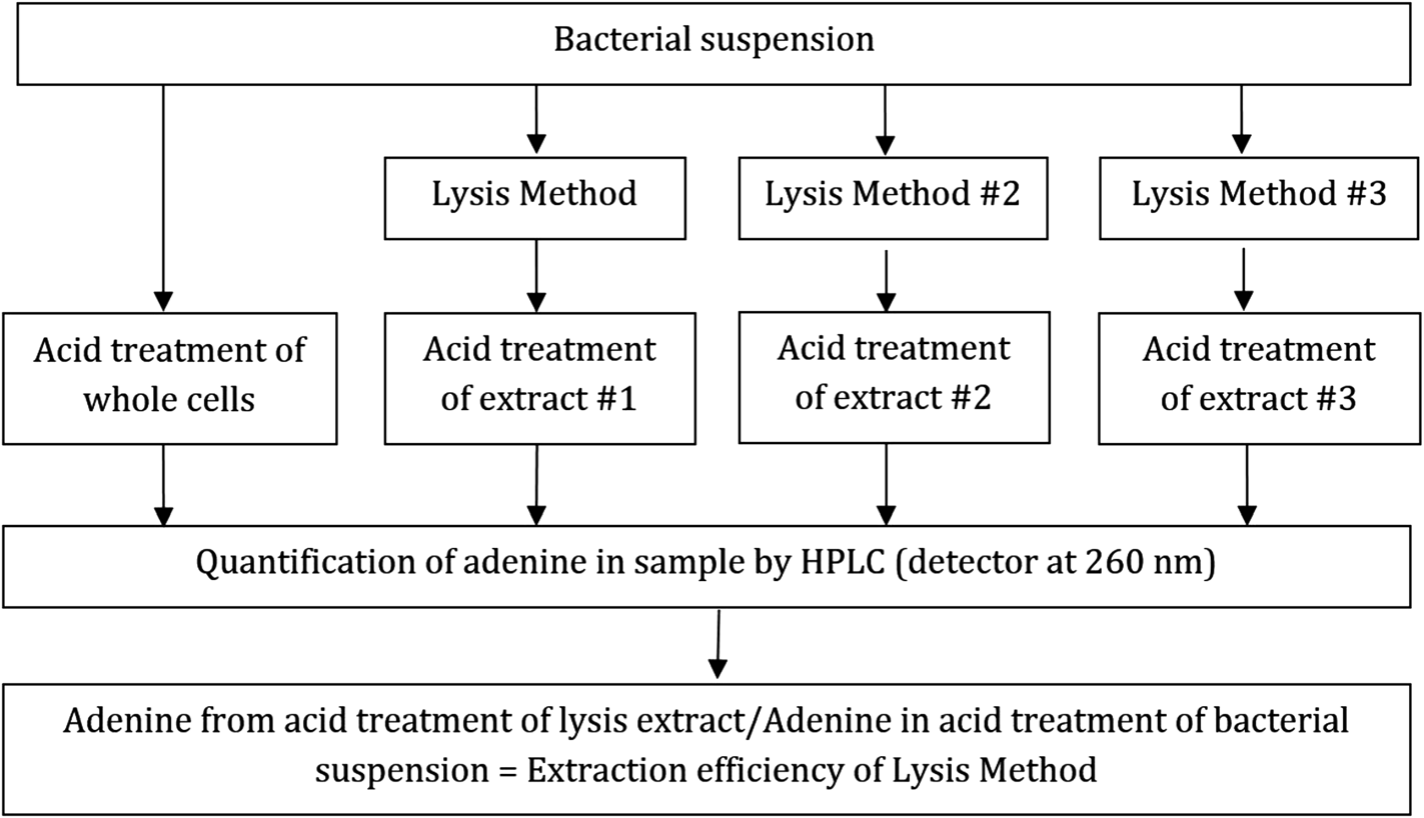
Schema showing use of the acid/HPLC method to determine DNA extraction efficiency by different methods. Equal aliquots of a bacterial suspension are centrifuged and the washed pellets are treated with standard acid/alkali or subjected to an extraction method (e.g., method #1. method #2, etc.). The extract is then treated by acid/alkali. The amount of adenine in the extract is compared to the amount of adenine from the pellet of intact cells. The efficiency of DNA extraction is given by the ratio of adenine (in extract) divided by adenine (from intact cells)

**Fig. 5:**
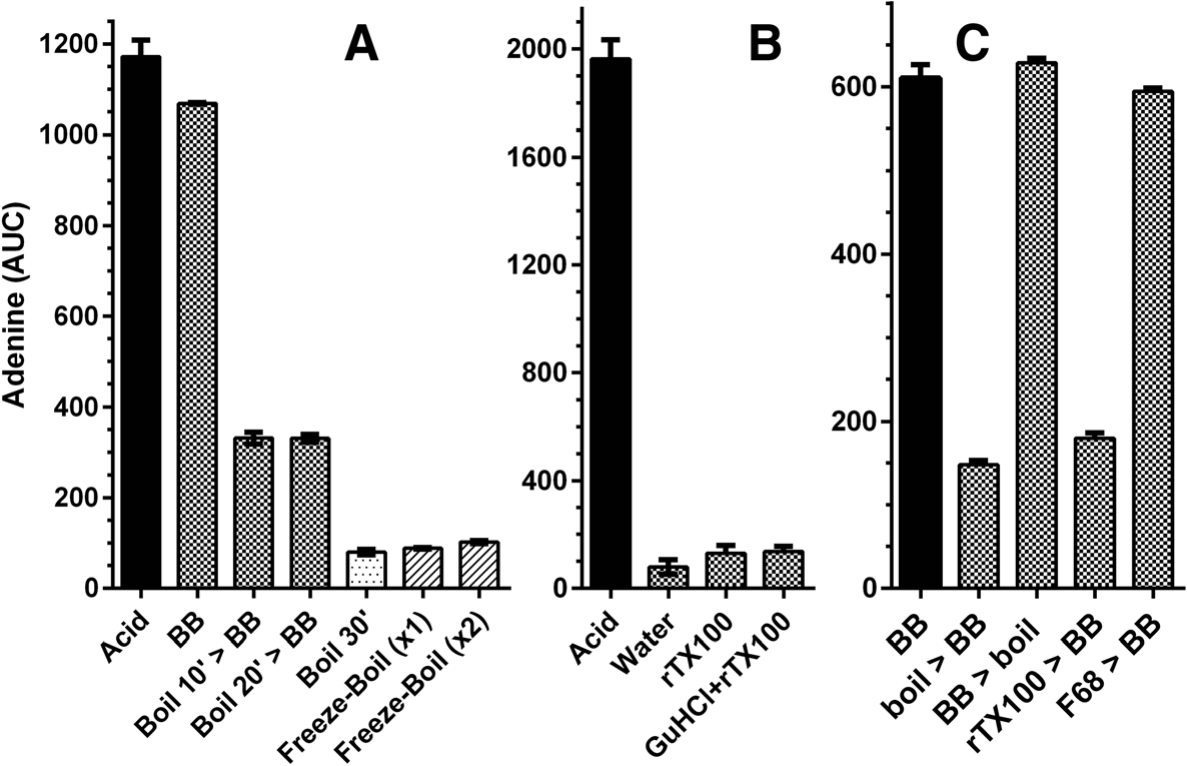
Comparison of the efficiency of DNA release from *M. smegmatis* cells by procedures in current use. A and B are results from two separate experiments. *Acid* in A and B indicates standard treatment with acid/alkali. **a** DNA release from 6×10^9^ *M. smegmatis* by bead-beating and boiling. > initial treatment was followed by a second treatment as indicated after the symbol. *BB*, bead-beating; *SDS + BB*, bead-beating of cells suspended in 1.0 % SDS; *boil*, suspension heated at 100 °C for the indicated time; *Freeze-boil*, cell suspension frozen at −20 °C, then heated at 100 °C for 10 min; ×2, cycle repeated once. **b** DNA release from 9×10^9^ *M. smegmatis* by guanidine hydrochloride. Cell suspension heated at 100 °C for 10 min in: *GuHCl + rTX100*, 8 M guanidine hydrochloride, 2 % reduced triton X-100, 80 mM Tris–HCl, 40 mM CDTA, pH 8.0; *rTX100*, 2 % reduced triton X-100, 80 mM Tris–HCl, 40 mM CDTA, pH 8.0; *Water*, water. **c** DNA released from *M. smegmatis* by bead-beating in combination with boiling or detergents. *BB*, bead-beating; *boil*, suspension heated at 100 °C for 10 min; *rTX100*, 2 % reduced triton X-100; 1 % *F68,* pluronic F-68. See Methods for additional details. Error bars represent the mean ± range of duplicate samples. Both ‘*rT100’* and *‘boil > BB’* are significantly different from *‘BB’* (*P* < 0.05, unpaired *t*-test)

Having demonstrated that the acid/HPLC method is suitable for quantifying DNA in intact bacteria, we used it to assess the efficiency of DNA release by a variety of extraction methods. An overview of the approach is shown in Fig. 4. For this experiment, we chose *M. smegmatis* as a target because it is a generally accepted model of a difficult-to-lyse bacterium. Bead-beating is a mechanical disruption step included in many DNA extraction protocols from *Mycobacterium* spp. As shown in Fig. 5a, bead-beating released about 90 % of the DNA. However, when bead-beating was preceded by heating at 100 °C for 10 min, only about 30 % of the DNA was released. The order of treatment mattered; when bead-beating was followed by heating, there was no decrease in the amount of DNA released (Fig. 5c). There was a similar diminution in the amount of DNA released when beading-beating was carried out in the presence of SDS (Fig. 5a) and triton X (Fig. 5c), but no decrease in the presence of another detergent, pluronic F-68 (Fig. 5c). Heating alone at 100 °C for 30 min or 1–2 freeze-boil cycles released 7–8 % of the total DNA from *M. smegmatis* (Fig. 5a).

In the experiment shown in Fig. 5b, heating a suspension of *M. smegmatis* in water at 100 °C for 10 min released 4.0 % release of total DNA. Guanidine hydrochloride, a chaotropic agent commonly used for DNA extractions, is often used in combination with triton X-100 in *Mycobacterium* extraction protocols. Including these reagents improved extraction to 7.0 %. Surprisingly, the contribution of guanidine hydrochloride to lysis efficiency was negligible, since heating with triton X-100 alone released 6.6 % of the total DNA.

Overall, our results demonstrate that commonly used DNA extraction methods can vary greatly in their ability to release DNA from a difficult-to-lyse bacterium.

### The acid/HPLC method is applicable to a broad-range of bacteria

To demonstrate that the acid/HPLC method is applicable to a variety of bacterial species, we also treated whole cells of *Francisella philomiragia, Bacillus thuringiensis, Moraxella catarrhalis, Pseudomonas aeruginosa, Staphylococcus aureus,* and *M. smegmatis* with acid and alkali, as described above. The amount of adenine released from these samples was compared to the amount of DNA in extracts produced by bead-beating. Based upon results shown in Fig. 2, bead-beating is somewhat less than 100 % effective in disrupting cells, so extracts of bead-beaten bacteria were expected to contain less adenine than detected by acid/alkali treatment of whole bacteria. The DNA extraction efficiency of bead-beating for five different microorganisms ranged from 70 to 80 % (Table 2). These results are similar to those reported using other methods [7], supporting our assertion that the acid/HPLC method can be applied to a variety of microbial species.

**Table 2:** Comparison of adenine in bead-beaten cell extracts with adenine in intact cells^a^

Organism	Adenine in extracts of bead-beated cells^b^ (a)	Adenine in intact cells^b^ (b)	Extraction efficiency (a/b)
*Mycobacterium smegmatis*	299.0 +/− 22.4	394.0 +/− 5.4	75.9 %
*Staphylococcus aureus*	736.1 +/− 25.2	922.9 +/− 18.4	79.7 %
*Francisella philomiragia*	496.6+/− 10.2	597.1 +/− 20.0	83.1 %
*Bacillus thuringiensis*	179.6 +/− 12.4	258.6 +/− 1.0	69.5 %
*Moraxella catarrhalis*	299.0 +/− 18.0	394 +/− 5.4	75.9 %
*Pseudomonas aeruginosa*	501.0 +/− 20.0	624.9 +/− 4.9	80.2 %

^a^Adenine released from DNA after acid/alkali treatment of extracts or intact cells (from approximately 10^9^ cells), as described in Fig. 4

^b^Area Under Curve of adenine peak (see Methods). Mean values +/− range of two separate samples are shown

## Discussion

We describe a method based on simple chemical principles for determining the total amount of DNA (and RNA) in an initial microbial sample. Complete release of adenine from DNA with little release from RNA is key to the acid/HPLC method. Release of guanine is expected to be similar, but this has not been proven. Once released from intracellular DNA by mild acid treatment, adenine must then be released into the extracellular milieu to be detectable. As shown in Fig. 2 and Table 1, the amount of adenine detected following acid/alkali treatment of intact cells and of disrupted cells is essentially identical, providing evidence that the acid/alkali treatment is sufficient to render cells porous to adenine. The acid/HPLC method permits detection of both intracellular DNA as well as released DNA, avoiding errors associated with use of two different DNA quantification methods (see Fig. 4). Although acid/HPLC is relatively insensitive as a detection method compared to qPCR or fluorescent dyes, it can detect single- and double-stranded DNA equally well. Finally, it also allows the quantification of intracellular RNA in the same HPLC run.

In order to calculate the efficiency of any extraction procedure, knowing the initial amount of DNA (prior to extraction) is required (see Fig. 4). Currently, there exists no direct method for determining extraction efficiency, although indirect methods have been used. Colony counting is one such indirect method, but it lacks precision because the amount of DNA per genome, the number of genomes per cell and the number of cells per CFU are difficult to ascertain. An alternative approach is to determine the initial amount of DNA by exposing cells to a treatment believed to completely lyse all cells, and then quantifying the liberated DNA. For example, enzymatic lysis of cells with lysozyme is very effective but is limited to lysozyme-sensitive, gram-positive bacteria. Although bead-beating is more universally applicable, lysis efficiency can be highly variable, dependent upon equipment, buffers and cell type. For spores and other difficult-to-disrupt cells, such as *Mycobacterium* spp., complete cell disruption is difficult to achieve using bead-beating [7]. Because the acid/HPLC method does not suffer from any of the above limitations, we believe that it has wide applicability.

The sensitivity of the method using HPLC with UV detection is 68 nanograms of adenine, or approximately 10^7^ cells. Other methods of detection, such as fluorescence and mass spectrometry, may decrease the number of cells required for analysis.

We provide several examples of the broad utility of the acid/HPLC method. We demonstrate that *B. subtilis* spores are relatively resistant to disruption by bead-beating, presumably due to their strong cell wall, small size and quasi-spherical shape. Calculating the amount of DNA in bacteria by colony counting significantly underestimates the amount of DNA in *B. subtilis* and *M. smegmatis*. The observed differences may be due to propensity of bacteria, particularly waxy microbes such as *M. smegmatis,* to form clumps. It should be noted, however, that colony counting measures only viable cells while the acid/HPLC method measures the DNA in both viable and nonviable cells. We demonstrate that minor differences in the bead-beating protocol for *M. smegmatis*, can produce large differences in the extraction efficiency (Fig. 5). Under optimal conditions, bead-beating was indeed an effective way to release DNA from these cells. However, when preceded by heat treatment (100 °C for 10–20 min), the efficiency of DNA release was markedly reduced. Reversing the order of treatments (bead-beating, then heating) did not reduce the effectiveness of bead-beating. This may be due to the formation of a flocculent suspension on heating. The presence of SDS, an anionic detergent commonly used in extraction protocols [21], produced a similar reduction in extraction efficiency. The likely cause is foaming, as a non-foaming detergent, pluronic F-68, did not affect extraction efficiency. These observations are of interest because bead-beating is often used in combination with heat treatment or SDS. The inefficiency of other methods for releasing DNA, such as heating in water at 100 °C, freeze-thawing or treatment with guanidine hydrochloride, was also demonstrated.

## Conclusions

Maximizing DNA extraction efficiency is a highly desirable goal for microbiome studies and for analyzing clinical samples. For microbiome studies, high extraction efficiency may increase the detection of difficult-to-lyse bacteria and the profile of extracted DNA may more closely resemble the actual profile of different species in the original sample. For clinical samples, high efficiency of extraction is expected to increase the likelihood of a correct diagnosis by recovering microbial DNA from hard-to-lyse organisms present in low numbers. It is our expectation that the acid/HPLC method will assist in the development of new, more efficient DNA extraction methods for use in these applications.

## Additional file

Additional file 1: Spreadsheet for converting nanomoles or nanograms of adenine to nanograms of DNA. (XLSX 17 kb)
